# Healthcare resources attributable to methicillin-resistant *Staphylococcus aureus* orthopedic surgical site infections

**DOI:** 10.1038/s41598-020-74070-4

**Published:** 2020-10-13

**Authors:** Haruhisa Fukuda, Daisuke Sato, Tetsuya Iwamoto, Koji Yamada, Kazuhiko Matsushita

**Affiliations:** 1 Department of Health Care Administration and Management, Kyushu University Graduate School of Medical Sciences, 3-1-1 Maidashi Higashi-ku, Fukuoka, 812-8582 Japan; 2 Center for Next Generation of Community Health, Chiba University Hospital, Chiba, Japan; 3 National Institute of Public Health, Saitama, Japan; 4 Department of Orthopaedic Surgery, Kanto Rosai Hospital, Kanagawa, Japan; 5 Department of Orthopaedic Surgery, Kawasaki Municipal Tama Hospital, Kanagawa, Japan

**Keywords:** Health care, Risk factors

## Abstract

The number of orthopedic surgeries is increasing as populations steadily age, but surgical site infection (SSI) rates remain relatively consistent. This study aimed to quantify the healthcare resources attributable to methicillin-resistant *Staphylococcus aureus* (MRSA) SSIs in orthopedic surgical patients. The analysis was conducted using a national claims database comprising data from almost all Japanese residents. We examined patients who underwent any of the following surgeries between April 2012 and March 2018: amputation (AMP), spinal fusion (FUSN), open reduction of fracture (FX), hip prosthesis (HPRO), knee prosthesis (KPRO), and laminectomy (LAM). Propensity score matching was performed to identify non-SSI control patients, and generalized estimating equations were used to estimate the differences in outcomes between the case and control groups. The numbers of MRSA SSI cases (infection rates) ranged from 64 (0.03%) to 1,152 (2.33%). MRSA SSI-attributable increases in healthcare expenditure ranged from $11,630 ($21,151 vs. $9,521) for LAM to $35,693 ($50,122 vs. $14,429) for FX, and increases in hospital stay ranged from 40.6 days (59.2 vs. 18.6) for LAM to 89.5 days (122.0 vs. 32.5) for FX. In conclusion, MRSA SSIs contribute to substantial increases in healthcare resource utilization, emphasizing the need to implement effective infection prevention measures for orthopedic surgeries.

## Introduction

Orthopedic surgeries are increasingly prevalent, with over 1 million primary total arthroplasties (both hip and knee)^1^ and 180,000 spine surgeries^2^ conducted in the US annually. In Japan, 195,000 primary total arthroplasties and 122,000 spine surgeries are performed every year^3^. Furthermore, the numbers of these surgeries are reportedly rising in the US^4^ and the UK^5^. Due to rapidly aging populations and longer life expectancies, orthopedic surgeries are likely to steadily increase in many countries.

Despite the high efficacy and success rates of orthopedic surgeries, a non-negligible proportion of these procedures require reoperations due to infection^6^. Although the infection rates in arthroplasty and spine surgeries are relatively low^4,7^, the occurrence of these infections can have a heavy impact on morbidity, mortality, and costs^8,9^. Surgical site infection (SSI) rates following arthroplasty have remained largely unchanged between 2005 and 2015^4^. In view of the growing volume of orthopedic surgeries, the economic impact of postoperative infections cannot be ignored. However, few studies have examined the additional use of healthcare resources required to treat orthopedic surgical patients with SSIs across a wide variety of medical institutions.

This study aimed to quantify the additional healthcare resources (i.e., healthcare expenditure and hospitalization duration) attributable to SSIs in orthopedic surgical patients in Japan using national-level data. The analysis focused on SSIs caused by methicillin-resistant *Staphylococcus aureus* (MRSA), which accounts for a large proportion of these infections in many countries.

## Results

The analysis was conducted using a total of 3,078,777 patients across the 6 target surgery types. The number of MRSA SSI cases and infection rates for each surgery type according to infection definition are presented in Table 1. When MRSA SSI cases were defined as patients who had been administered anti-MRSA antibiotics for 7 days or more and had undergone surgical debridement (i.e., the base case definition), the number of MRSA SSI cases (infection rate) for amputation (AMP), spinal fusion (FUSN), open reduction of fracture (FX), hip prosthesis (HPRO), knee prosthesis (KPRO), and laminectomy (LAM) were 1,152 (2.33%), 1,279 (0.23%), 1,046 (0.08%), 567 (0.09%), 257 (0.07%), and 64 (0.03%), respectively. With the exception of AMP, the SSI rates did not exceed 1% among the surgery types. When debridement was not included in this definition, infection rates were substantially increased by 7.01% (LAM) to 0.14% (AMP).

**Table 1:** Number of MRSA SSI cases (infection rates) according to infection definition.

MRSA SSI definition	AMP	FUSN	FX	HPRO	KPRO	LAM
Anti-MRSA antibiotics for ≥ 7 days with debridement (base case)	1,152 (2.33%)	1,279 (0.23%)	1,046 (0.08%)	567 (0.09%)	257 (0.07%)	64 (0.03%)
Anti-MRSA antibiotics for ≥ 5 days with debridement	1,245 (2.50%)	1,423 (0.26%)	1,163 (0.09%)	617 (0.10%)	284 (0.07%)	78 (0.04%)
Anti-MRSA antibiotics for ≥ 7 days	3,469 (7.01%)	4,657 (0.84%)	4,849 (0.38%)	3,436 (0.56%)	1,254 (0.33%)	272 (0.14%)
Anti-MRSA antibiotics for ≥ 5 days	3,837 (7.70%)	5,344 (0.96%)	5,569 (0.43%)	3,884 (0.64%)	1,406 (0.37%)	316 (0.16%)

AMP, amputation; FUSN, spinal fusion; FX, open reduction of fracture; HPRO, hip prosthesis; KPRO, knee prosthesis; LAM, laminectomy; MRSA, methicillin-resistant *Staphylococcus aureus*; SSI, surgical site infection.

The characteristics of patients with and without MRSA SSI before propensity score matching are summarized according to surgery type in Table 2. SSI patients tended to have higher Charlson comorbidity index (CCI) scores and higher proportions of men, older patients, diabetic patients requiring insulin, and patients requiring dialysis. The proportion of patients with rheumatic disease was high for all surgery types except AMP. In the propensity score-matched groups, the absolute values of the standardized difference were < 10% for all baseline characteristics (Table 3).

**Table 2:** Characteristics and unadjusted outcomes of patients with MRSA SSIs^a^ and without SSIs before propensity score matching.

	AMP (n = 16,921)			FUSN (n = 370,934)			FX (n = 856,404)		
	Non-SSI	MRSA SSI	*P* value	Non-SSI	MRSA SSI	*P* value	Non-SSI	MRSA SSI	*P* value
Women, n (%)	6,264 (39.7%)	357 (31.0%)	< 0.001	153,574 (41.6%)	403 (31.5%)	< 0.001	602,885 (70.5%)	525 (50.2%)	< 0.001
Age: 0–64 y	3,366 (21.4%)	374 (32.5%)	< 0.001	119,013 (32.2%)	354 (27.7%)	< 0.001	245,908 (28.8%)	329 (31.5%)	< 0.001
Age: 65–74 y	3,828 (24.3%)	332 (28.8%)		124,865 (33.8%)	400 (31.3%)		138,052 (16.1%)	195 (18.6%)	
Age: 75–84 y	4,789 (30.4%)	299 (26.0%)		111,029 (30.0%)	425 (33.2%)		223,357 (26.1%)	263 (25.1%)	
Age: ≥ 85 y	3,786 (24.0%)	147 (12.8%)		14,748 (4.0%)	100 (7.8%)		248,041 (29.0%)	259 (24.8%)	
No diabetes	9,685 (61.4%)	639 (55.5%)		289,226 (78.2%)	796 (62.2%)		715,147 (83.6%)	719 (68.7%)	
Diabetes: recorded diagnosis	1,439 (9.1%)	33 (2.9%)	< 0.001	29,784 (8.1%)	89 (7.0%)	< 0.001	43,988 (5.1%)	62 (5.9%)	< 0.001
Diabetes: antidiabetic agent prescription	922 (5.9%)	51 (4.4%)		12,080 (3.3%)	55 (4.3%)		29,738 (3.5%)	30 (2.9%)	
Diabetes: insulin administration	3,723 (23.6%)	429 (37.2%)		38,565 (10.4%)	339 (26.5%)		66,485 (7.8%)	235 (22.5%)	
Dialysis treatment	12,009 (76.2%)	715 (62.1%)	< 0.001	364,685 (98.7%)	1,206 (94.3%)	< 0.001	845,797 (98.9%)	993 (94.9%)	< 0.001
Rheumatic disease	3,760 (23.8%)	437 (37.9%)	0.278	4,970 (1.3%)	73 (5.7%)	< 0.001	9,561 (1.1%)	53 (5.1%)	< 0.001
CCI, mean [SD]	15,373 (97.5%)	1,129 (98.0%)	< 0.001	362,813 (98.2%)	1,212 (94.8%)	< 0.001	841,565 (98.4%)	1,011 (96.7%)	< 0.001
LOS, day	396 (2.5%)	23 (2.0%)	< 0.001	6,842 (1.9%)	67 (5.2%)	< 0.001	13,793 (1.6%)	35 (3.4%)	< 0.001
Healthcare expenditure, US$	1.7 [1.8]	2.2 [1.9]	< 0.001	0.5 [1.1]	1.7 [2.5]	< 0.001	0.8 [1.4]	1.6 [2.2]	< 0.001

	HPRO (n = 372,094)			KPRO (n = 230,506)			LAM (n = 154,652)		
	Non-SSI	MRSA SSI	*P* value	Non-SSI	MRSA SSI	*P* value	Non-SSI	MRSA SSI	*P* value
Women, n (%)	303,059 (81.6%)	337 (59.4%)	< 0.001	185,789 (80.7%)	179 (69.7%)	< 0.001	53,273 (34.5%)	13 (20.3%)	0.017
Age: 0–64 y	81,716 (22.0%)	85 (15.0%)	< 0.001	21,940 (9.5%)	34 (13.2%)	0.002	106,950 (69.2%)	35 (54.7%)	0.018
Age: 65–74 y	89,518 (24.1%)	103 (18.2%)		78,891 (34.3%)	66 (25.7%)		28,836 (18.7%)	17 (26.6%)	
Age: 75–84 y	120,568 (32.5%)	201 (35.5%)		113,083 (49.1%)	129 (50.2%)		16,746 (10.8%)	n.r	
Age: ≥ 85 y	79,725 (21.5%)	178 (31.4%)		16,335 (7.1%)	28 (10.9%)		2,056 (1.3%)	n.r	
No diabetes	310,720 (83.6%)	373 (65.8%)		181,244 (78.7%)	171 (66.5%)		133,878 (86.6%)	45 (70.3%)	
Diabetes: recorded diagnosis	19,486 (5.2%)	50 (8.8%)	< 0.001	19,311 (8.4%)	22 (8.6%)	< 0.001	9,800 (6.3%)	n.r	< 0.001
Diabetes: antidiabetic agent prescription	10,817 (2.9%)	25 (4.4%)		9,065 (3.9%)	20 (7.8%)		2,973 (1.9%)	n.r	
Diabetes: insulin administration	30,504 (8.2%)	119 (21.0%)		20,629 (9.0%)	44 (17.1%)	< 0.001	7,937 (5.1%)	13 (20.3%)	
Dialysis treatment	366,413 (98.6%)	519 (91.5%)	< 0.001	229,139 (99.5%)	251 (97.7%)	< 0.001	154,052 (99.7%)	62 (96.9%)	< 0.001
Rheumatic disease	5,114 (1.4%)	48 (8.5%)	< 0.001	1,110 (0.5%)	n.r	< 0.001	536 (0.4%)	n.r	0.032
CCI, mean [SD]	360,266 (97.0%)	533 (94.0%)	< 0.001	214,803 (93.3%)	226 (87.9%)	< 0.001	153,390 (99.2%)	62 (96.9%)	< 0.001
LOS, day	11,261 (3.0%)	34 (6.0%)	< 0.001	15,446 (6.7%)	31 (12.1%)	< 0.001	1,198 (0.8%)	n.r	< 0.001
Healthcare expenditure, US$	0.7 [1.3]	2.3 [2.4]	< 0.001	0.4 [0.9]	1.4 [1.9]	< 0.001	0.2 [0.7]	0.7 [1.2]	< 0.001

^a^MRSA SSI cases were identified as those prescribed anti-MRSA antibiotics for ≥ 7 days and had undergone surgical debridement.

AMP, amputation; CCI, Charlson comorbidity index; FUSN, spinal fusion; FX, open reduction of fracture; HPRO, hip prosthesis; KPRO, knee prosthesis; LAM, laminectomy; LOS, length of stay; MRSA, methicillin-resistant *Staphylococcus aureus*; SD, standard deviation; SSI, surgical site infection; n.r., not reportable in accordance with the data dissemination standards of the NDB.

**Table 3:** Characteristics of patients with and without MRSA SSIs^a^ after propensity score matching.

	AMP			FUSN			FX			HPRO			KPRO			LAM		
	Non-SSI (n = 1151)	MRSA SSI (n = 1151)	SD	Non-SSI (n = 1279)	MRSA SSI (n = 1279)	SD	Non-SSI (n = 1046)	MRSA SSI (n = 1046)	SD	Non-SSI (n = 567)	MRSA SSI (n = 567)	SD	Non-SSI (n = 257)	MRSA SSI (n = 257)	SD	Non-SSI (n = 64)	MRSA SSI (n = 64)	SD
Women, n (%)	366 (31.8%)	357 (31.0%)	− 0.017	398 (31.1%)	403 (31.5%)	0.008	521 (49.8%)	525 (50.2%)	0.008	330 (58.2%)	337 (59.4%)	0.025	180 (70.0%)	179 (69.7%)	− 0.008	14 (21.9%)	13 (20.3%)	− 0.038
Age: 0–64 y	368 (32.0%)	373 (32.4%)	0.009	354 (27.7%)	354 (27.7%)	0.000	324 (31.0%)	329 (31.5%)	0.010	82 (14.5%)	85 (15.0%)	0.015	33 (12.8%)	34 (13.2%)	0.012	35 (54.7%)	35 (54.7%)	0.000
Age: 65–74 y	338 (29.4%)	332 (28.8%)	− 0.011	389 (30.4%)	400 (31.3%)	0.019	200 (19.1%)	195 (18.6%)	− 0.012	104 (18.3%)	103 (18.2%)	− 0.005	67 (26.1%)	66 (25.7%)	− 0.009	15 (23.4%)	17 (26.6%)	0.072
Age: 75–84 y	301 (26.2%)	299 (26.0%)	− 0.004	436 (34.1%)	425 (33.2%)	− 0.018	261 (25.0%)	263 (25.1%)	0.004	201 (35.5%)	201 (35.5%)	0.000	129 (50.2%)	129 (50.2%)	0.000	n.r	n.r	− 0.044
Age: ≥ 85 y	144 (12.5%)	147 (12.8%)	0.008	100 (7.8%)	100 (7.8%)	0.000	261 (25.0%)	259 (24.8%)	− 0.004	180 (31.8%)	178 (31.4%)	− 0.008	28 (10.9%)	28 (10.9%)	0.000	n.r	n.r	− 0.069
No diabetes	632 (54.9%)	638 (55.4%)	0.010	794 (62.1%)	796 (62.2%)	0.003	723 (69.1%)	719 (68.7%)	− 0.008	373 (65.8%)	373 (65.8%)	0.000	175 (68.1%)	171 (66.5%)	− 0.033	44 (68.8%)	45 (70.3%)	0.034
Diabetes: recorded diagnosis	30 (2.6%)	33 (2.9%)	0.016	86 (6.7%)	89 (7.0%)	0.009	64 (6.1%)	62 (5.9%)	− 0.008	49 (8.6%)	50 (8.8%)	0.006	19 (7.4%)	22 (8.6%)	0.043	n.r	n.r	0.000
Diabetes: antidiabetic agent prescription	50 (4.3%)	51 (4.4%)	0.004	56 (4.4%)	55 (4.3%)	− 0.004	29 (2.8%)	30 (2.9%)	0.006	23 (4.1%)	25 (4.4%)	0.018	19 (7.4%)	20 (7.8%)	0.015	n.r	n.r	− 0.069
Diabetes: insulin administration	439 (38.1%)	429 (37.3%)	− 0.018	343 (26.8%)	339 (26.5%)	− 0.007	230 (22.0%)	235 (22.5%)	0.011	122 (21.5%)	119 (21.0%)	− 0.013	44 (17.1%)	44 (17.1%)	0.000	13 (20.3%)	13 (20.3%)	0.000
Dialysis treatment	434 (37.7%)	436 (37.9%)	0.004	73 (5.7%)	73 (5.7%)	0.000	53 (5.1%)	53 (5.1%)	0.000	48 (8.5%)	48 (8.5%)	0.000	n.r	n.r	− 0.048	n.r	n.r	n.a
Rheumatic disease	16 (1.4%)	23 (2.0%)	0.047	59 (4.6%)	67 (5.2%)	0.029	35 (3.4%)	35 (3.4%)	0.000	28 (4.9%)	34 (6.0%)	0.047	27 (10.5%)	31 (12.1%)	0.049	n.r	n.r	0.000
CCI, mean [SD]	2.1 [1.8]	2.2 [1.9]	0.030	1.7 [2.5]	1.7 [2.5]	0.000	1.6 [2.2]	1.6 [2.2]	− 0.002	2.2 [2.4]	2.3 [2.4]	0.012	1.4 [1.9]	1.4 [1.9]	− 0.012	0.7 [1.2]	0.7 [1.2]	0.000

^a^MRSA SSI cases were identified as those prescribed anti− MRSA antibiotics for ≥ 7 days and had undergone surgical debridement.

AMP, amputation; FUSN, spinal fusion; FX, open reduction of fracture; HPRO, hip prosthesis; KPRO, knee prosthesis; LAM, laminectomy; MRSA, methicillin− resistant *Staphylococcus aureus*; SD, standard deviation; SD, standardized difference; SSI, surgical site infection; n.r., not reportable in accordance with the data dissemination standards of the NDB.

Table 4 shows the results of the regression analysis with MRSA SSI occurrence (base case) as the dependent variable. In all 6 surgery types, the analysis identified men, diabetes status, dialysis treatment, and CCI score to be significantly associated with MRSA SSI occurrence. Furthermore, rheumatic disease was significantly associated with MRSA SSI occurrence for FUSN, FX, and HPRO. Among FUSN patients, the month of surgery was associated with MRSA SSI occurrence, where surgeries in May to October had odds ratios of 1.48 to 1.84. Among HPRO patients, May, July, and August had odds ratios of 1.63, 1.58, and 1.72, respectively, for MRSA SSI occurrence.

**Table 4:** Factors associated with MRSA SSI^a^ occurrence: results of logistic regression analyses.

	AMP	FUSN	FX	HPRO	KPRO	LAM
	OR (95% CI)	OR (95% CI)	OR (95% CI)	OR (95% CI)	OR (95% CI)	OR (95% CI)
Women	0.91 (0.80–1.05)	0.66 (0.58–0.74)***	0.45 (0.39–0.51)***	0.42 (0.35–0.50)***	0.62 (0.47–0.81)**	0.48 (0.26–0.88)*
Age: 0–64 y	1.30 (1.11–1.52)**	1.10 (0.95–1.27)	1.10 (0.91–1.33)	1.10 (0.82–1.47)	1.76 (1.16–2.67)**	0.74 (0.41–1.36)
Age: 65–74 y	REF	REF	REF	REF	REF	REF
Age: 75–84 y	0.83 (0.70–0.97)**	1.21 (1.05–1.38)**	0.80 (0.66–0.96)*	1.27 (1.00–1.61)	1.29 (0.96–1.75)	0.91 (0.40–2.04)
Age: ≥ 85 y	0.68 (0.55–0.85)***	2.11 (1.69–2.64)***	0.81 (0.67–0.98)*	1.70 (1.33–2.18)***	1.83 (1.17–2.86)**	2.44 (0.71–8.45)
No diabetes	REF	REF	REF	REF	REF	REF
Diabetes: recorded diagnosis	0.62 (0.43–0.90)*	1.07 (0.86–1.33)	1.34 (1.03–1.75)*	1.85 (1.37–2.50)***	1.06 (0.68–1.66)	0.78 (0.24–2.55)
Diabetes: antidiabetic agent prescription	1.49 (1.09–2.05)*	1.50 (1.14–1.98)**	1.01 (0.70–1.47)	1.67 (1.11–2.52)**	2.05 (1.28–3.27)**	2.28 (0.69–7.51)
Diabetes: insulin administration	2.91 (2.44–3.48)***	2.92 (2.56–3.33)***	3.48 (2.98–4.06)***	2.80 (2.26–3.47)***	2.01 (1.43–2.81)***	3.82 (1.99–7.32)***
Dialysis treatment	2.62 (2.18–3.14)***	3.56 (2.78–4.55)***	3.46 (2.59–4.63)***	4.27 (3.10–5.87)***	3.08 (1.35–7.06)**	5.55 (1.24–24.74)*
Rheumatic disease	1.06 (0.68–1.63)	2.27 (1.76–2.92)***	1.95 (1.39–2.75)***	1.85 (1.30–2.64)**	1.25 (0.85–1.85)	2.76 (0.64–12.00)
CCI	1.12 (1.09–1.16)***	1.36 (1.33–1.39)***	1.25 (1.22–1.29)***	1.35 (1.31–1.39)***	1.48 (1.40–1.58)***	1.37 (1.14–1.64)**
Surgery month: January	REF	REF	REF	REF	REF	REF
Surgery month: February	0.89 (0.67–1.18)	1.19 (0.87–1.63)	0.86 (0.64–1.17)	1.11 (0.72–1.71)	0.82 (0.41–1.67)	9.31 (1.19–72.81)*
Surgery month: March	0.81 (0.62–1.07)	1.20 (0.88–1.63)	0.78 (0.57–1.07)	1.02 (0.66–1.58)	1.01 (0.51–1.97)	3.39 (0.38–30.33)
Surgery month: April	0.85 (0.64–1.13)	1.25 (0.91–1.71)	0.97 (0.72–1.32)	1.26 (0.82–1.94)	1.14 (0.58–2.24)	4.88 (0.57–41.77)
Surgery month: May	1.02 (0.77–1.34)	1.61 (1.19–2.17)**	1.16 (0.87–1.54)	1.63 (1.09–2.45)*	1.99 (1.09–3.63)*	4.88 (0.57–41.76)
Surgery month: June	0.86 (0.65–1.15)	1.48 (1.10–1.99)**	1.35 (1.02–1.79)*	1.44 (0.94–2.20)	1.83 (1.02–3.30)*	9.61 (1.24–74.48)
Surgery month: July	0.92 (0.69–1.23)	1.84 (1.39–2.45)***	1.21 (0.91–1.62)	1.58 (1.05–2.39)*	1.48 (0.80–2.74)	5.94 (0.73–48.30)
Surgery month: August	0.95 (0.71–1.28)	1.65 (1.23–2.21)**	1.31 (0.98–1.74)	1.72 (1.14–2.58)**	1.24 (0.64–2.42)	4.41 (0.51–37.76)
Surgery month: September	0.86 (0.62–1.18)	1.65 (1.23–2.22)**	1.22 (0.92–1.64)	1.37 (0.89–2.11)	1.41 (0.75–2.65)	3.75 (0.42–33.56)
Surgery month: October	0.96 (0.71–1.30)	1.78 (1.33–2.37)***	0.97 (0.73–1.31)	1.30 (0.85–1.97)	1.35 (0.73–2.52)	4.39 (0.51–37.59)
Surgery month: November	0.81 (0.59–1.11)	1.17 (0.85–1.59)	1.26 (0.96–1.67)	1.30 (0.86–1.98)	1.58 (0.86–2.89)	1.90 (0.17–20.97)
Surgery month: December	1.01 (0.76–1.36)	1.22 (0.89–1.67)	0.98 (0.74–1.31)	1.37 (0.91–2.07)	1.21 (0.60–2.46)	4.45 (0.52–38.10)

* < 0.05, ** < 0.01, *** < 0.001.

^a^MRSA SSI cases were identified as those prescribed anti-MRSA antibiotics for ≥ 7 days and had undergone surgical debridement.

AMP, amputation; CCI, Charlson comorbidity index; CI, confidence interval; FUSN, spinal fusion; FX, open reduction of fracture; HPRO, hip prosthesis; KPRO, knee prosthesis; LAM, laminectomy; MRSA, methicillin-resistant *Staphylococcus aureus*; OR, odds ratio; SSI, surgical site infection.

The estimates of MRSA SSI–attributable healthcare expenditure and length of hospital stay (LOS) from the generalized estimating equations after propensity score matching are presented in Table 5. In all surgery types, MRSA SSIs were significantly associated with increases in healthcare expenditure and LOS. The analysis estimated MRSA SSI–attributable increases in healthcare expenditure of $33,290 ($53,003 vs. $19,713) for AMP, $28,858 ($48,461 vs. $19,603) for FUSN, $35,693 ($50,122 vs. $14,429) for FX, $24,252 ($47,784 vs. $23,532) for HPRO, $24,667 ($47,120 vs. $22,453) for KPRO, and $11,630 ($21,151 vs. $9,521) for LAM. The analysis also estimated MRSA SSI–attributable increases in LOS of 69.4 days (118.6 vs. 49.2) for AMP, 61.1 days (90.4 vs. 29.3) for FUSN, 89.5 days (122.0 vs. 32.5) for FX, 75.0 days (118.1 vs. 43.1) for HPRO, 74.9 days (115.1 vs. 40.2) for KPRO, and 40.6 days (59.2 vs. 18.6) for LAM.

**Table 5:** Estimates of outcomes in patients with and without MRSA SSIs from generalized estimating equations after propensity score matching.

	Healthcare expenditure			Length of stay		
	Non-SSI	MRSA SSI	*P* value	Non-SSI	MRSA SSI	*P* value
**AMP**						
Anti-MRSA antibiotics for ≥ 7 days with debridement (base case)	19,713 [499]	53,003 [1337]	< 0.001	49.2 [1.8]	118.6 [4.3]	< 0.001
Anti-MRSA antibiotics for ≥ 5 days with debridement	19,629 [471]	52,725 [1264]	< 0.001	49.5 [1.7]	117.6 [4.0]	< 0.001
Anti-MRSA antibiotics for ≥ 7 days	20,646 [322]	45,337 [707]	< 0.001	53.3 [1.2]	105.3 [2.4]	< 0.001
Anti-MRSA antibiotics for ≥ 5 days	20,629 [316]	44,451 [680]	< 0.001	53.4 [1.2]	102.9 [2.2]	< 0.001
**FUSN**						
Anti-MRSA antibiotics for ≥ 7 days with debridement (base case)	19,603 [302]	48,461 [746]	< 0.001	29.3 [0.7]	90.4 [2.0]	< 0.001
Anti-MRSA antibiotics for ≥ 5 days with debridement	20,043 [309]	47,221 [729]	< 0.001	29.2 [0.6]	87.8 [1.8]	< 0.001
Anti-MRSA antibiotics for ≥ 7 days	20,048 [164]	45,091 [370]	< 0.001	30.2 [0.4]	81.7 [1.0]	< 0.001
Anti-MRSA antibiotics for ≥ 5 days	19,527 [149]	43,780 [335]	< 0.001	29.0 [0.3]	78.1 [0.8]	< 0.001
**FX**						
Anti-MRSA antibiotics for ≥ 7 days with debridement (base case)	14,429 [268]	50,122 [930]	< 0.001	32.5 [0.8]	122.0 [3.2]	< 0.001
Anti-MRSA antibiotics for ≥ 5 days with debridement	14,305 [265]	49,912 [925]	< 0.001	32.9 [0.9]	123.6 [3.4]	< 0.001
Anti-MRSA antibiotics for ≥ 7 days	15,999 [150]	41,271 [386]	< 0.001	39.0 [0.5]	112.3 [1.6]	< 0.001
Anti-MRSA antibiotics for ≥ 5 days	15,975 [144]	39,937 [359]	< 0.001	39.1 [0.6]	108.3 [1.6]	< 0.001
**HPRO**						
Anti-MRSA antibiotics for ≥ 7 days with debridement (base case)	23,532 [486]	47,784 [986]	< 0.001	43.1 [1.6]	118.1 [4.3]	< 0.001
Anti-MRSA antibiotics for ≥ 5 days with debridement	22,548 [411]	47,140 [859]	< 0.001	38.7 [1.2]	114.6 [3.5]	< 0.001
Anti-MRSA antibiotics for ≥ 7 days	22,746 [211]	42,751 [395]	< 0.001	41.4 [0.7]	101.1 [1.6]	< 0.001
Anti-MRSA antibiotics for ≥ 5 days	22,745 [194]	41,632 [355]	< 0.001	41.0 [0.6]	97.4 [1.3]	< 0.001
**KPRO**						
Anti-MRSA antibiotics for ≥ 7 days with debridement (base case)	22,453 [634]	47,120 [1333]	< 0.001	40.2 [1.7]	115.1 [5.0]	< 0.001
Anti-MRSA antibiotics for ≥ 5 days with debridement	20,901 [551]	45,357 [1197]	< 0.001	34.9 [1.4]	109.4 [4.5]	< 0.001
Anti-MRSA antibiotics for ≥ 7 days	21,111 [293]	39,493 [549]	< 0.001	34.9 [0.7]	85.1 [1.8]	< 0.001
Anti-MRSA antibiotics for ≥ 5 days	21,280 [283]	38,296 [509]	< 0.001	35.2 [0.7]	82.0 [1.7]	< 0.001
**LAM**						
Anti-MRSA antibiotics for ≥ 7 days with debridement (base case)	9,521 [378]	21,151 [835]	< 0.001	18.6 [1.3]	59.2 [4.0]	< 0.001
Anti-MRSA antibiotics for ≥ 5 days with debridement	9,312 [354]	20,314 [767]	< 0.001	18.6 [1.2]	55.9 [3.7]	< 0.001
Anti-MRSA antibiotics for ≥ 7 days	10,658 [310]	29,416 [851]	< 0.001	21.4 [0.9]	78.9 [3.4]	< 0.001
Anti-MRSA antibiotics for ≥ 5 days	10,707 [290]	27,590 [744]	< 0.001	22.0 [0.8]	73.4 [2.8]	< 0.001

Values are presented as mean [standard deviation].

AMP, amputation; FUSN, spinal fusion; FX, open reduction of fracture; HPRO, hip prosthesis; KPRO, knee prosthesis; LAM, laminectomy; MRSA, methicillin-resistant *Staphylococcus aureus*; SSI, surgical site infection.

The annual budget impact analysis (Table 6) estimated that MRSA SSIs in orthopedic surgeries were associated with additional expenditures of $6.39 million for AMP, $6.15 million for FUSN, $6.22 million for FX, $2.29 million for HPRO, $1.06 million for KPRO, and $120,000 for LAM. The total budget impact for all 6 procedures was $22.24 million.

**Table 6:** Annual budget impact of MRSA SSI in orthopedic surgery.

	Attributable expenditure ($ per MRSA SSI case)	Number of MRSA SSI cases per year	Budget impact per year, million $		
			Base case	Lower	Upper
AMP	33,290	192	6.39	5.84	6.94
FUSN	28,858	213	6.15	5.81	6.49
FX	35,693	174	6.22	5.89	6.56
HPRO	24,252	95	2.29	2.08	2.50
KPRO	24,667	43	1.06	0.93	1.18
LAM	11,630	11	0.12	0.10	0.14
Total			22.24	20.66	23.82

AMP, amputation; FUSN, spinal fusion; FX, open reduction of fracture; HPRO, hip prosthesis; KPRO, knee prosthesis; LAM, laminectomy; MRSA, methicillin-resistant *Staphylococcus aureus*; SSI, surgical site infection.

## Discussion

In an analysis of national-level claims data, this study quantified the economic impact of MRSA SSIs in over 3 million patients across 6 types of orthopedic surgery. Orthopedic surgeries have relatively low SSI rates, and few studies have produced quantitative estimates of the additional healthcare resources attributable to these infections. The estimates presented in this study therefore provide new evidence from (to the best of our knowledge) the largest dataset of orthopedic surgeries to date.

For studies that quantify MRSA SSI–attributable increases in healthcare expenditure, the use of patients with antibiotic-susceptible infections (“replacement” model where resistant infections replace susceptible infections in the total disease burden) versus the use of patients without infections (“addition” model where resistant infections add to the total disease burden) as the comparison group is a point of debate^10–12^. This lack of consensus among researchers is exemplified in the variety of approaches used to explore the disease burden of antibiotic-resistant infections, with some studies using the replacement model^13,14^, some using the addition model^15–17^, and others using a combination of both^18,19^. This study utilizes the addition model with a control group comprising non-SSI patients. In another study conducted in Japan, Uematsu et al. reported that MRSA infections had significantly higher healthcare expenditures than patients with methicillin-susceptible *S. aureus* infections using the replacement model^20^. While the existing literature contains studies that have utilized either approach, we consider that both strategies have value in contributing to our understanding of the disease burden of antibiotic-resistant infections, and support the development of effective measures against disease transmission and the improvement of antibiotic use to reduce the selection of resistant bacterial strains. In this way, our study provides valuable data for the addition model.

This study represents, to the authors’ knowledge, the first report from Japan that examines MRSA SSI occurrence, risk factors, and attributable healthcare expenditure in a variety of orthopedic surgery types in Japan. Previous studies in Japan have addressed the antimicrobial susceptibility of pathogens^21^, trends in reoperation for MRSA SSIs in spinal surgery patients only^22^, SSI incidence in orthopedic surgery patients^23^, and additional healthcare expenditures attributable to MRSA infections not limited to SSIs^20,24^. However, these studies have not focused on MRSA SSIs in orthopedic surgery patients. Furthermore, a strength of this study is that separate analyses were conducted for 6 different orthopedic surgery types, which provides insight into the differential impact of MRSA SSI among these procedures.

As this study was conducted without data on microbiological test results and SSI surveillance, MRSA SSI cases were identified using information on anti-MRSA antibiotics available in claims data. This method of analyzing MRSA cases is similar to that used in Uematsu et al., which examined the additional expenditures associated with MRSA infections^24^. As a consequence, the MRSA cases identified in this study may also include infections by methicillin-resistant *Staphylococcus epidermidis* (MRSE) and coagulase-negative staphylococci (CNS). The Japanese government established the Japan Nosocomial Infections Surveillance (JANIS) program in 2000 to monitor the incidence and prevalence of nosocomial infections in voluntary participant hospitals. According to that program, the rates of deep incisional and organ/space SSIs caused by MRSA, MRSE, and CNS in 2017 were 0.20% for AMP, 0.31% for FUSN, 0.10% for FX, 0.08% for HPRO, 0.08% for KPRO, and 0.25% for LAM^25^. While our SSI rates were similar to these estimates for FUSN, FX, HPRO, and KPRO, our estimated SSI rate for AMP was 10 times that reported by the JANIS program. Many AMP patients undergo amputations for gangrene of the lower leg due to infections or ischemia, and a large proportion of these patients have severe infections prior to surgery^26^. Therefore, our antibiotic-based SSI identification method may have included patients who had been receiving long-term antibiotic treatment that continued after surgery. Our analysis may also have underestimated SSI rates for LAM. When compared with the other surgery types, LAM patients without infection tended to have short LOS durations below 30 days, which may have caused us to overlook SSIs that occurred after being discharged. Systematic reviews have estimated *S. aureus* SSI rates in the US and Europe to be 1.7% and 0.6%, respectively, after arthroplasty^8^ and 1.9% and 1.0%, respectively, after spine surgery^9^. However, the possible inclusion of non-MRSA infections in our subjects makes direct comparisons of these rates problematic. Nevertheless, when considering that MRSA accounts for 40–50% of *S. aureus* infections^9^ and that our MRSA SSI cases may also include MRSE and CNS infections, our estimated MRSA SSI rates are likely to be lower than those reported in the US and Europe.

After propensity score matching, the healthcare expenditure attributable to MRSA SSIs was 2.03 (HPRO) to 3.47 (FX) times more than the control patients, and the additional LOS attributable to MRSA SSIs was 2.41 (AMP) to 3.75 (FX) times more than the control patients. Several studies have reported that patients with SSIs have healthcare expenditures that are 1.4–3.7 times more than that of uninfected patients after arthroplasty^8^, but few of these studies have focused on MRSA infections. Edwards et al. compared the additional healthcare expenditures between 39 MRSA cases and 41 non-MRSA controls, and found that the expenditure for the cases was only 1.19 times (*P* = 0.964) that of the controls^27^. However, it should be noted that their findings were susceptible to influence by the small sample size. In addition, few studies have compared healthcare expenditures between patients with and without SSIs after spine surgery. Kuhns et al. reported that the healthcare expenditure of SSI cases was 2.29 times that of non-SSI controls among spine surgical patients^28^, but that study was a single-institutional analysis that did not take into consideration the causative pathogens. Accordingly, our study is the first to use large-scale data to quantify the additional healthcare expenditure attributable to MRSA SSIs in orthopedic surgical patients.

The findings of this study should be interpreted with consideration to the following limitations. First, MRSA SSIs were identified through the use of anti-MRSA antibiotic prescription patterns. Japanese guidelines recommend the discontinuation of antimicrobial prophylaxis within 24 or 48 h (depending on surgery type) after orthopedic surgery^29^. Therefore, our use of a 7-day cut-off for anti-MRSA antibiotic administration reduces the likelihood of misidentifying cases with routine prophylaxis as those being treated for MRSA infections. However, these antibiotics may have targeted infections other than SSI, and we sought to more accurately identify SSI cases through our database by including surgical debridement for the base case analysis. In addition, anti-MRSA antibiotics are also used to treat MRSE and CNS SSI cases, which may have been included in our subjects. According to the JANIS program, the proportions of MRSA SSIs among all methicillin-resistant staphylococcal SSIs are 100% for AMP, 72.8% for FUSN, 80.2% for FX, 77.1% for HPRO, 60.0% for KPRO, and 60.5% for LAM^25^. Second, we only analyzed infections that occurred during the index hospitalization. Although the US Centers for Disease Control and Prevention recommends a 90-day follow-up period after orthopedic surgery to detect SSIs^30^, patients who developed SSIs after being discharged were not included in our analysis. However, due to the relatively long hospitalizations for non-SSI patients in Japan (as shown in Table 5), we expect that a large proportion of SSIs would be detected during the initial hospitalization.

## Conclusions

This study analyzed an unprecedented number of patients to quantify the potential economic burden of MRSA SSIs after orthopedic surgery. As the world’s population continues to age, orthopedic surgeries are expected to become increasingly common. Although these surgeries are associated with relatively low infection rates, MRSA SSIs can have an inordinately heavy clinical and economic impact. Our findings indicate a need to explore further measures to prevent SSIs after orthopedic surgeries. As this study was conducted using almost all eligible patients in Japan, our estimates may also have applications when investigating the cost-effectiveness of such measures.

## Methods

### Study design

**Figure 1:**
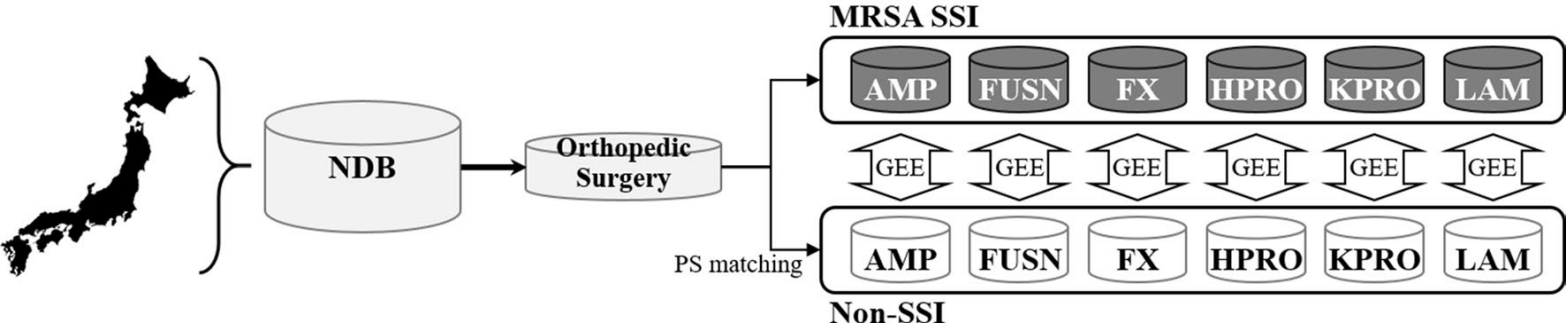
Schematic illustration of the study design. The Database of Health Insurance Claims and Specific Health Checkups of Japan (NDB) collects and stores data from hospitals throughout Japan. From this database, we obtained data on patients who had been hospitalized for any of the following orthopedic surgery types between April 1, 2012 and March 31, 2018: amputation (AMP), spinal fusion (FUSN), open reduction of fracture (FX), hip prosthesis (HPRO), knee prosthesis (KPRO), and laminectomy (LAM). The patients were then divided into those with methicillin-resistant *Staphylococcus aureus* (MRSA) surgical site infections (SSI) as the case group and those without any SSI (non-SSI) as the control group. The 2 groups underwent propensity score (PS) matching, and generalized estimating equations (GEEs) were used to estimate their differences in health expenditure and length of stay.

Using a national-level claims database, this study employed a retrospective case–control design to quantify the differences in healthcare expenditure and LOS between orthopedic surgical patients with MRSA SSIs (i.e., cases) and those without any SSIs (i.e., controls). Control patients were identified using propensity score matching (Fig. 1).

The study was approved by the Kyushu University Institutional Review Board for Clinical Research (Approval Number 30–149), which acts in accordance with the ethical standards laid down in the “non-interventional clinical research” in Japan. The Kyushu University Institutional Review Board for Clinical Research also allowed the researchers to waive the requirement for obtaining informed consent because of the retrospective nature of the study and the use of anonymized data.

### Database

Data were obtained from the National Database of Health Insurance Claims and Specific Health Checkups of Japan (NDB). The NDB collects and anonymizes all insurance claims data sent from medical institutions to insurers for the purpose of reimbursement, and provides anonymized datasets to authorized researchers for analysis after an application and review process. As Japan utilizes a universal health insurance system, the database encompasses data from all Japanese residents who use healthcare services covered by insurance. The claims data include detailed information on recorded diagnoses on admission, surgical procedures, pharmaceuticals, and healthcare expenditure, but lack clinical information regarding diagnostic testing and vital signs. The study subjects were identified using surgical procedures and prescription patterns, and the designated outcomes were compared between the case and control patients.

### Patients

The subjects comprised patients who had been hospitalized for orthopedic surgery between April 1, 2012 and March 31, 2018. We analyzed the following 6 orthopedic surgery types: AMP, FUSN, FX, HPRO, KPRO, and LAM. Based on the *International Classification of Diseases, Ninth Revision, Clinical Modification* codes employed by the US National Healthcare Safety Network^31^, each surgery type was identified using the corresponding Japanese surgical procedure codes.

To examine patients with primary surgery and exclude cases of recent reoperations, we limited the analysis to patients who had not undergone any of the 6 target surgery types between April 2010 and March 2012. In addition, patients who underwent any of the 6 target surgery types after the index surgery were excluded from analysis.

### Definition of MRSA SSI and Non-SSI

The NDB does not include results of blood tests, diagnostic microbiological tests, or antimicrobial susceptibility tests. As an alternative method to identify MRSA SSI cases, we examined the postoperative prescriptions of anti-MRSA antibiotics in each patient. Bolon et al. have previously identified SSI cases as patients who were administered antibiotics for 7 days or more after surgery^32^. Based on this criterion, we analyzed prescriptions of linezolid, vancomycin, teicoplanin, arbekacin, and daptomycin, which represent anti-MRSA antibiotics that are covered under insurance in Japan. To more accurately identify SSI cases, the base case analysis focused on patients who had also undergone surgical debridement.

As a sensitivity analysis, we also identified MRSA SSI cases as those who were administered anti-MRSA antibiotics for 5 days or more after surgery. Furthermore, not all SSI cases would have undergone surgical debridement. Therefore, this study identified MRSA SSI cases using the following 4 definitions: (1) patients administered anti-MRSA antibiotics for 7 days or more and had undergone surgical debridement, (2) patients administered anti-MRSA antibiotics for 5 days or more and had undergone surgical debridement, (3) patients administered anti-MRSA antibiotics for 7 days or more, and (4) patients administered anti-MRSA antibiotics for 5 days or more. The first definition was used in the base case analysis, and the remaining 3 definitions were used in the sensitivity analysis.

To estimate the differences in healthcare resources used between the MRSA SSI cases and non-SSI controls, we excluded all patients with suspected SSIs (including those with non-MRSA SSIs) from the latter. Specifically, we identified and excluded all orthopedic surgical patients who were administered any antibiotic for 5 days or more after surgery or had undergone surgical debridement from the control group.

### Outcomes

The primary outcome was healthcare expenditure during hospitalization for orthopedic surgery. Healthcare expenditure in this study referred to the amount billed by the medical institutions to the insurers for the provision of healthcare to each patient, and therefore represents costs from the insurer’s perspective. In Japan, the fee schedule for medical goods and services undergoes minor revisions every 2 years, and our study period included 3 periods (2012–2013, 2014–2015, and 2016–2018) in which the unit prices may vary. For this analysis, all unit prices were adjusted to 2018 prices by applying the revision rates for each year of data. Japanese yen were converted to US dollars using the purchasing power parity rate in 2017 ($1.00 = 102.5 yen). The secondary outcome was LOS, which was calculated as the number of days from the date of admission to the date of discharge for the index hospitalization.

### Statistical analysis

We first identified MRSA SSI cases among the patients hospitalized for orthopedic surgery between April 1, 2012 and March 31, 2018. Corresponding non-SSI controls were then identified using propensity score matching. Propensity scores were calculated for each of the 4 definitions of MRSA SSI using logistic regression models with SSI occurrence or non-occurrence as the dependent variable. The independent variables included diabetes status, dialysis treatment, rheumatic disease, sex, age, month of surgery, and CCI score. Several meta-analyses have identified diabetes status as a risk factor of SSI^33–35^. In this study, we analyzed 4 different statuses: no diabetes, recorded diabetes diagnosis, prescription of antidiabetic agents, and administration of insulin. The use or non-use of dialysis was also examined. Similarly, rheumatic disease has been previously reported to be an SSI risk factor^33,34^, and was identified in this study using the relevant recorded diagnoses in the claims data. As seasonal variability has been reported to be a risk factor of SSI^36–39^, each patient’s month of surgery was included in the regression models as a dummy variable. We then performed 1:1 matching with non-SSI control patients without replacement using a caliper width of 0.25 standard deviations of the logit of each propensity score. Standardized differences were used to test for balances between the case and control groups.

When analyzing healthcare resource utilization, a proportion of cases with inordinately high expenditures or protracted hospitalizations may shift the distribution of utilization such that it resembles a gamma distribution instead of a normal distribution^40,41^. If calculating the differences in mean healthcare resource utilization between the case and control groups with the assumption of a normal distribution, the presence of such outliers may introduce bias into the estimates. Alternatively, modal values can be used, but these would not provide insight into the mean healthcare resource utilization attributable to infections, and are not suitable for use in national-level budget impact analyses. As a result, the utilization of the case and control groups were estimated after propensity score matching using generalized estimating equations under the assumption of a gamma distribution. These estimates were used to calculate the mean healthcare expenditure and LOS attributable to MRSA SSIs.

All statistical analyses were performed using Stata/MP version 15.1 (Stata Corp., College Station, Texas, USA). *P* values (two-tailed) below 0.05 were considered statistically significant.

### Budget impact

To estimate the total direct medical costs of MRSA SSI in orthopedic surgeries to Japan’s healthcare system, we quantified the annual budget impact of these infections. The budget impact was estimated using the total number of MRSA SSI base cases (i.e., those administered anti-MRSA antibiotics for 7 days or more and had undergone surgical debridement). As the study was conducted using 6 years of data, the annual budget impact was calculated using the mean annual number of cases and the MRSA SSI–attributable healthcare expenditure estimates produced in the main analysis. As a sensitivity analysis, we calculated the lower and upper limits of the MRSA SSI–attributable healthcare expenditures using the mean ± 2 standard deviations.

## Data Availability

The data that support the findings reported here were obtained from the Japanese Ministry of Health, Labour and Welfare under license for the current study, and are not publicly available due to restrictions stipulated by the ministry.
